# Perceived esthetics and value of clear aligner therapy systems: A survey among dental school instructors and undergraduate students

**DOI:** 10.1590/2177-6709.28.3.e232225.oar

**Published:** 2023-07-24

**Authors:** Christos LIVAS, Fatema Shabnam PAZHMAN, Zeynep ILBEYLI, Nikolaos PANDIS

**Affiliations:** 1 Dental Clinics Zwolle, Division of Orthodontics (Zwolle, The Netherlands).; 2 University of Amsterdam and VU University Amsterdam, Academic Centre for Dentistry Amsterdam (ACTA), Master Dentistry program (Amsterdam, The Netherlands).; 3 University of Bern, School of Dental Medicine, Department of Orthodontics and Dentofacial Orthopaedics (Bern, Switzerland).

**Keywords:** Clear aligner therapy, Esthetics, Dental professionals, Practice management

## Abstract

**Objective:**

To investigate the attractiveness, acceptability, visibility and willingness-to-pay for clear aligner therapy (CAT) systems in first-year and final-year dental students and instructors.

**Methods:**

A questionnaire designed to collect information regarding esthetic preferences and intentions related to seven CAT systems was handed out to 120 undergraduate students and instructors at the Academic Centre for Dentistry Amsterdam (ACTA). Proportional odds models and population average generalized estimating equation models were used to examine potential association between participant characteristics, esthetic perceptions and CAT systems.

**Results:**

Overall, the examined CAT systems received favorable esthetic ratings. Expertise status was significantly associated with willingness-to-pay additionally for CAT, compared to fixed orthodontic appliances. There was no association between sex, previous orthodontic treatment history, satisfaction with own dental appearance and potential interest in treatment and aligner visibility and willingness-to-pay. CAT system was significantly associated with the perceived aligner visibility, acceptability and attractiveness by students and instructors.

**Conclusions:**

CAT systems were considered to a great extent attractive and acceptable for future treatment by dental school instructors and students. Willingness-to-pay for CAT systems was significantly associated with expertise status, with instructors appearing more reluctant to pay for CAT.

## RESUMO

**Objetivo:**

Comparar diferentes sistemas de tratamento com alinhadores transparentes (CAT), quanto à atratividade, aceitabilidade, visibilidade e disposição a pagar, por parte de alunos (primeiro e último anos) e instrutores de Odontologia.

**Métodos:**

Um questionário elaborado para coletar informações sobre preferências e intenções estéticas, em relação a sete sistemas CAT, foi distribuído para 120 alunos de graduação e instrutores do Academic Centre for Dentistry Amsterdam (ACTA). Modelos de riscos proporcionais e modelos de equação de estimação generalizada para a média da população foram usados para examinar a possível associação entre as características dos participantes, percepções estéticas e os sistemas CAT.

**Resultados:**

No geral, os sistemas CAT examinados receberam avaliações estéticas favoráveis. O nível de experiência foi significativamente associado com a disposição em pagar mais por sistemas CAT do que por aparelhos ortodônticos fixos. Não houve associação entre sexo, histórico de tratamento ortodôntico anterior, satisfação com a própria aparência dentária, potencial interesse em tratamento, visibilidade do alinhador e disposição em pagar mais. Os sistemas CAT foram significativamente associados à visibilidade percebida, aceitabilidade e atratividade dos alinhadores por alunos e instrutores.

**Conclusões:**

Os sistemas CAT foram considerados, em grande parte, atraentes e aceitáveis para tratamentos futuros pelos instrutores e alunos do curso de Odontologia. A disposição em pagar mais pelos sistemas CAT foi significativamente associada ao nível de especialização, com os instrutores parecendo mais relutantes em pagar mais pelo CAT.

## INTRODUCTION

Public awareness regarding dental appearance has been intensified over the years. Facial and dental attractiveness has been associated with high social competence, intellectual achievement, and favorable psychological development.^1^ On the contrary, malocclusion features such as irregular tooth position or inter-arch relationship may negatively affect the perception of overall attractiveness and well-being.^2^ Claimed psychosocial effects of dental esthetics may prompt individuals to seek orthodontic care.^3^

The rising impact of dental esthetics on social perceptions has raised the demands for adult orthodontics.^4^ According to data from the British Orthodontic Society, three quarters of the registered orthodontists have reported an increase of adult private patients.^5^ However, orthodontic appliance design and appearance may influence decision to initiate treatment and appliance preference.^6^ ^,^ ^7^ Thirty-three to 62% of adults would decline treatment with visible orthodontic appliances because of poor esthetics.^8^ ^,^ ^9^ To reduce appliance visibility, more esthetically attractive treatment appliances and accessories have emerged, including plastic and ceramic brackets, tooth-coloured wires, lingual brackets and clear aligners. 

Clear Aligner Therapy (CAT), originally based on Kesling’s tooth positioning device,^10^ became worldwide popular among clinicians and patients when Invisalign aligners (Align Technology, Inc., San Jose, CA, USA) were introduced as a viable treatment alternative to fixed appliances. Nowadays, more than 27 different CAT products are commercially available,^11^ while nearly 9 out of 10 practices in USA routinely perform treatment with clear aligners.^12^

Despite the widespread CAT growth, the perceived attractiveness of clear aligners has been rarely investigated. Fixed appliances with colored elastic ties were classified by children as more attractive than clear aligners.^13^ ^,^ ^14^ In contrast, adults rated clear aligners and lingual brackets more favorably compared to ceramic and metallic brackets.^15^ ^,^ ^16^ Moreover, lay adults were willing to pay significantly more for less visible appliances such as lingual appliances and clear aligners for themselves and their children.^15^

Study populations in the above-mentioned studies^13^ ^-^ ^16^ comprised laypersons of a broad age range, lacking dental expertise. Given the varying influence of education level and clinical experience on esthetics assessment, this study aimed to investigate the attractiveness, acceptability, visibility and value of CAT systems in dental school instructors and undergraduate students. 

## METHODS

### CAT SYSTEM INCLUSION

Following a Google search (https://www.google.com/) using the term ‘clear aligners’, the first five pages were screened for eligible systems. Manufacturers were contacted by e-mail or by filling the contact form displayed on the company’s website. Additionally, domestic orthodontic laboratories fabricating in-house aligners were reached by e-mail and phone. Five aligner companies (ClearCorrect^TM^, Dentsply Sirona, Modern Me GmbH, Orthocaps Gmbh, Ortholab B.V.) agreed to supply free aligner samples. Orthocaps Gmbh contributed with three aligner products, i.e., one made of single-layer polymer (SLP), and two made of double-layer polymer (DLP). In total, seven CAT systems were investigated for the purposes of the study (Table 1).

**Table 1:** Manufacturer and origin details of the CAT systems examined in the study.

CAT system	Manufacturer	Origin
ClearCorrect^TM^ aligner	ClearCorrect	Round Rock, TX, USA
Ideal Smile^®^ ALIGNER	Dentsply Sirona	York, PA, USA
MODERN CLEAR system	Modern Me GmbH	Düsseldorf, Germany
Orthocaps^®^ SLP 800	Ortho Caps GmbH	Hamm, Germany
Orthocaps^®^ DLP 460		
Orthocaps^®^ DLP 580		
Ortho Aligner	Ortholab B.V.	Doorn, The Netherlands

### CAT SYSTEM FABRICATION AND PHOTOGRAPHIC TECHNIQUE

Digital impressions of a consenting female dental student were obtained using TRIOS^®^ dental intraoral scanner (3shape, Copenhagen, Denmark). The selection criteria were: well-aligned dental arches and lack of strong sex markers in the circumoral region.^14^ Scanned data were exported as .STL files and e-mailed to the collaborating aligner manufacturers. 

**Figure 1:**
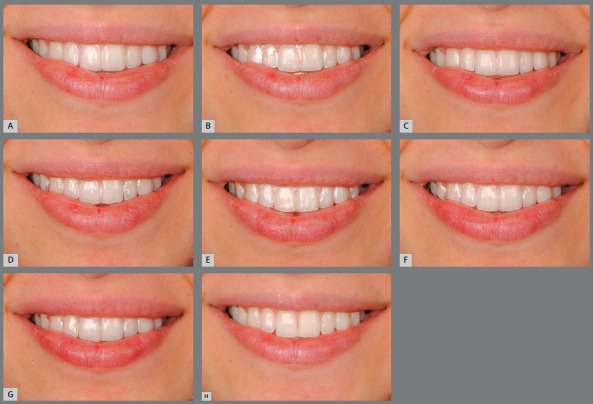
Standardized images of the volunteer with (**A-G**) and without (H) CAT systems: A) ClearCorrect^TM^ aligner; B) MODERN CLEAR system; C) Ortho Aligner; D) Ideal Smile^®^ ALIGNER; E) Orthocaps^®^ SLP 800; F) Orthocaps^®^ DLP 460; G) Orthocaps^®^ DLP 580.

Smiling coloured photographs of the volunteer with and without the aligners (i.e., 8 images in total) were captured with a digital camera, a Nikon D3000 (Nikon Corporation, Tokyo, Japan) with an AF Micro Nikkon lens 105mm. The camera was equipped with a Sigma EM-140DG flash set to ¼ power. All images were taken under the same conditions in JPEG format on manual settings adjusted to F stop 20, shutter speed 1/160 and ISO100. Image standardization for color and format was performed with Photoshop CC (version 19.1.3, Adobe, San Jose, CA, US). To ensure the true-life size of the images, the mesiodistal width of the maxillary central incisor was fixed at 8 mm.^16^ Figure 1 presents the standardized images acquired by the photographic technique of the study.

### SAMPLE RECRUITMENT AND QUESTIONNAIRE DESIGN

Ethical approval for this survey was granted from the Ethics Committee of the Academish Centrum Tandheelkunde Amsterdam (ACTA; protocol number, 2018063). All participants were either students (first- and sixth-year students) or dentists employed as clinical instructors at ACTA, willing to participate in the survey. Before enrolling, each participant was informed about the research objectives, instructed on how to complete the survey, and signed an informed consent. 

**Figure 2:**
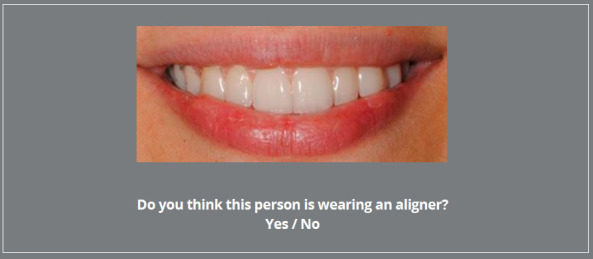
Survey question regarding CAT system visibility.

Based on previous studies on appliance esthetics,^15^ ^,^ ^16^ a two-part questionnaire was developed. The first part consisted of questions related to demographics (i.e., sex, age, professional expertise), and orthodontic treatment aspects (i.e., orthodontic treatment history, interest in undergoing orthodontic treatment in the future, satisfaction with own dental appearance, and potential willingness to pay more for CAT, compared to conventional metallic brackets).Visibility, attractiveness, and acceptability of the aligners were determined in the second part, using images displayed in random order and coupled with image rating questions. At first, participants were asked to confirm the presence or absence of aligners on standardized smiling images (Fig 2).

**Figure 3:**
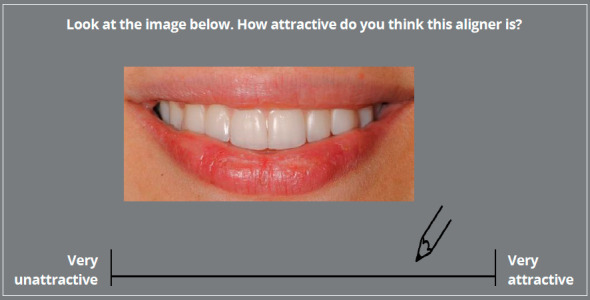
Survey question regarding CAT system attractiveness.

To rate aligner attractiveness, a Visual Analogue Scale (VAS) question was applied to aligner images twice, randomly displayed. The random sequences, i.e., E-A-F-C-H-B-D-G and C-G-F-E-A-B-D-H, were created with an online random sequence generator (https://www.random.org/). The VAS scale had a length of 100 mm and was anchored by “very unattractive” and “very attractive”. Each participant marked on the scale indicating his/her perception of attractiveness (Fig 3). One observer (second author) measured the distance between the “very unattractive”-end and the mark, using a digital caliper (Digital Caliper U-59112, FINO GmbH, Bad Bocklet, Germany) reading up to two decimal places. Finally, a yes-or-no question was included to rate aligner acceptability by asking participants if they would be willing to wear the given CAT system in a hypothetical orthodontic treatment (Fig 3).

## STATISTICAL ANALYSIS

Descriptive statistics for the baseline patient characteristics overall and according to the levels of willingness to pay and visibility were calculated. For the outcome willingness to pay, univariable proportional odds models were fit to examine potential associations with participant characteristics. A multivariable proportional odds model was fit, that included the significant variables from the first model. For the outcome visibility, univariable population average generalized estimating equation (GEE) models with empirical standard errors were fit to examine potential associations with participant characteristics. A multivariable population average GEE model with empirical standard errors was fit, that included the significant variables from the first model. For the effect of CAT on the acceptability and attractiveness population, average GEE models with empirical standard errors were fit. The GEE models were used to account for the correlated data, resulting from the fact that the same participants were used for all the CAT systems. All analyses were conducted using Stata 16.1 (Stata Corp, TX, USA) and R software version 3.6.1 (R Foundation for Statistical Computing, Vienna, Austria), with a two-sided 5% level of statistical significance.

## RESULTS

### SAMPLE CHARACTERISTICS

Forty first-year students, 40 sixth-year students and 39 instructors completed the survey. The majority of the participants were females (57.98%), previously orthodontically treated (63.90%), and potentially interested in future treatment (52.90%, Table 2). 

**Table 2:** Descriptive statistics of the participants’ characteristics.

	Mean (SD)		Range (years)
Age	30.8 (13.9)		17-66
		n	%
Sex	Males	50	42.02
	Females	69	57.98
Expertise status	First-year students	40	33.61
	Sixth-year students	40	33.61
	Instructors	39	32.78
Orthodontic treatment history	Yes	76	63.86
	No	43	36.14
Interest in future treatment	Yes	63	52.94
	No	48	47.06
Satisfaction with own dental esthetics	Yes	93	78.15
	No	22	21.85

### WILLINGNESS-TO-PAY

**Figure 4:**
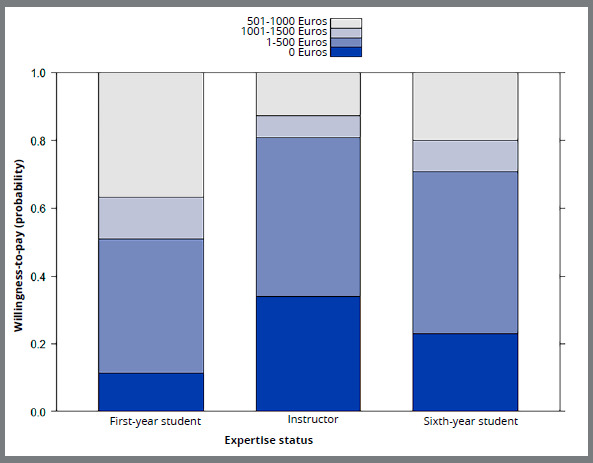
Predicted probabilities for willingness-to-pay, per expertise status.

More than 76% of the participants were willing to pay an additional amount to receive CAT instead of conventional fixed orthodontics appliances, mainly up to 500 Euros (Table 3). Fewer instructors intended to pay for clear aligners compared to first-year and last-year students, i.e., 54.05% vs. 85% and 91.18%, respectively. Previously treated participants willing to pay additionally for CAT systems were 2.15 times as many as those not treated. Students and instructors satisfied with their dental appearance were more eager in paying more for clear aligners, compared to dissatisfied peers, i.e., 77.91% vs. 71.43%, respectively. Participants interested in future treatment showed a greater willingness-to-pay for CAT than those without interest, and vice versa (Table 3). In the univariable analysis (Table 4), there was no association between willingness-to-pay and sex, previous orthodontic treatment history, and satisfaction with own dental appearance. Expertise status and interest in future treatment were associated with willingness to pay for clear aligners, but only expertise status remained a strong intention-to-pay predictor in the multivariable analysis (Table 4). In particular, the odds for instructors to pay an additional amount to receive CAT in the future were 72% lower, compared to first-year year students. Figure 4 shows the predicted probabilities for willingness to pay per expertise status, as obtained from the multivariable GEE model. 

**Table 3:** Distribution of participants’ willingness-to-pay responses and CAT system visibility responses per group.

	Willingness-to-pay				Visibility			
Sex	0 Euros	1-500 Euros	501-1000 Euros	1001-1500 Euros	Total	Visible	Not visible	Total
Male	15 (57.70%)	15 (32.60%)	11 (40.70%)	6 (50.00%)	47 (42.30%)	193 (40.40%)	207 (43.70%)	400 (42.00%)
Female	11 (42.30%)	31 (67.40%)	16 (59.30%)	6 (50.00%)	64 (57.70%)	285 (59.60%)	267 (56.30%)	552 (58.00%)
Total	26 (100%)	46 (100%)	27 (100%)	12 (100%)	111 (100%)	478 (100%)	474 (100%)	952 (100%)
Expertise status	0 Euros	1-500 Euros	501-1000 Euros	1001-1500 Euros	Total	Visible	Not visible	Total
First-year students	3 (11.50%)	15 (32.60%)	14 (51.90%)	5 (41.70%)	37 (33.30%)	154 (32.20%)	166 (35.00%)	320 (33.60%)
Sixth-year students	9 (34.60%)	18 (39.10%)	9 (33.30%)	4 (33.30%)	40 (36.00%)	148 (31.00%)	172 (36.30%)	320 (33.60%)
Instructors	14 (53.80%)	13 (28.30%)	4 (14.80%)	3 (25.00%)	34 (30.60%)	176 (36.80%)	136 (28.70%)	312 (32.80%)
Total	26 (100%)	46 (100%)	27 (100%)	12 (100%)	111 (100%)	478 (100%)	474 (100%)	952 (100%)
Orthodontic treatment history	0 Euros	1-500 Euros	501-1000 Euros	1001-1500 Euros	Total	Visible	Not visible	Total
Yes	15 (57.70%)	29 (63.00%)	20 (74.10%)	9 (75.00%)	73 (65.80%)	190 (39.70%)	154 (32.50%)	344 (36.10%)
No	11 (42.30%)	17 (37.00%)	7 (25.90%)	3 (25.00%)	38 (34.20%)	288 (60.30%)	320 (67.50%)	608 (63.90%)
Total	26 (100%)	46 (100%)	27 (100%)	12 (100%)	111 (100%)	478 (100%)	474 (100%)	952 (100%)
Satisfaction with own dental esthetics	0 Euros	1-500 Euros	501-1000 Euros	1001-1500 Euros	Total	Visible	Not visible	Total
Yes	19 (76.00%)	36 (83.70%)	25 (92.60%)	6 (50.00%)	86 (80.40%)	371 (80.50%)	373 (81.30%)	744 (80.90%)
No	6 (24.00%)	7 (16.30%)	2 (7.40%)	6 (50.00%)	21 (19.60%)	90 (19.50%)	86 (18.70%)	176 (19.10%)
Total	25 (100%)	43 (100%)	27 (100%)	12 (100%)	107 (100%)	461 (100%)	459 (100%)	920 (100%)
Interest in future treatment	0 Euros	1-500 Euros	501-1000 Euros	1001-1500 Euros	Total	Visible	Not visible	Total
Yes	8 (33.30%)	29 (65.90%)	19 (73.10%)	7 (70.00%)	63 (60.60%)	207 (46.50%)	177 (40.00%)	384 (43.20%)
No	16 (66.70%)	15 (34.10%)	7 (26.90%)	3 (30.00%)	41 (39.40%)	238 (53.50%)	266 (60.00%)	504 (56.80%)
Total	24 (100%)	44 (100%)	26 (100%)	10 (100%)	104 (100%)	445 (100%)	443 (100%)	888 (100%)

**Table 4:** Univariable and multivariable proportional odds regression model results for the effect of participant characteristics on willingness-to-pay for CAT systems.

	Univariable			Multivariable		
	Odds Ratio	p-value	95% CI	Odds Ratio	p-value	95% CI
Sex						
Male	reference					
Female	1.28	0.49	0.64, 0.53			
Expertise status						
1st-year students	reference			reference		
6th-year students	0.47	0.07	0.21, 1.06	0.50	0.12	0.21, 1.18
Instructors	0.21	<0.01	0.09, 0.53	0.28	0.01	0.11, 0.74
Treatment history						
no	reference					
yes	1.70	0.15	0.83, 3.51			
Satisfaction with own dental esthetics						
no	reference					
yes	1.22	0.68	0.48, 3.12			
Interest in treatment						
no	reference			reference		
yes	2.92	0.01	1.36, 6.26	2.15	0.07	0.95, 4.87

### VISIBILITY

Females, instructors, earlier orthodontically treated or participants interested in future treatment were more capable of identifying CAT systems on the photographs, compared to males, students and those without experience or interest in treatment (Table 3). The distribution of visibility responses depending on presence of CAT system are tabulated in Table 5. According to the univariable analysis results, aligner visibility was associated with expertise status treatment history, interest in future orthodontic treatment and CAT system. However, in the multivariable analysis, only the CAT system remained a significant predictor (Table 6).

### ACCEPTABILITY

The distribution of acceptability responses per CAT system is tabulated in Table 5. Five CAT systems were found acceptable for future treatment by more than 83% of the participants (Table 5). CAT system was significantly associated with acceptability (*p*<0.001, Table 7).

**Table 5:** Distribution of CAT system visibility and acceptability responses, and attractiveness scores.

CAT system	Visibility		Acceptability		Attractiveness score	
	not visible	visible	not acceptable	acceptable		
ClearCorrect^TM^ aligner						
n	96	23	19	97	Mean	68.91
%	80.67	19.33	16.38	83.62	SD	15.75
MODERN CLEAR system						
n	24	95	12	104	Mean	71.80
%	20.17	79.83	10.34	89.66	SD	13.45
Ortho Aligner						
n	89	30	12	105	Mean	72.39
%	74.79	25.21	10.26	89.74	SD	13.79
Ideal Smile^®^ ALIGNER						
n	78	41	14	101	Mean	73.15
%	65.55	34.45	12.17	87.83	SD	15.05
Orthocaps^®^ SLP 800						
n	6	113	86	32	Mean	34.78
%	5.04	94.96	72.88	27.12	SD	19.24
Orthocaps^®^ DLP 460						
n	44	75	13	103	Mean	68.89
%	36.97	63.03	11.21	88.79	SD	14.30
Orthocaps^®^ DLP 580						
n	24	95	35	83	Mean	63.04
%	20.17	79.83	29.66	70.34	SD	17.44
No aligner						
n	113	6				
%	94.96	5.04				

**Table 6:** Univariable and multivariable population average GEE regression model results for the effect of participant characteristics on CAT system visibility.

	Univariable			Multivariable		
	Odds Ratio	***p*-value**	95% CI	Odds Ratio	***p*-value**	95% CI
Sex						
Male	reference					
Female	0.15	0.317	0.88, 1.49			
Expertise status						
1st-year students	reference			reference		
6th-year students	0.93	0.65	0.67, 1.28	0.82	0.49	0.48, 1.42
Instructors	1.39	0.05	1.00, 1.94	1.56	0.15	0.86, 2.85
Treatment history						
No	reference			reference		
Yes	0.73	0.02	0.56, 0.95	0.77	0.27	0.48, 1.23
Satisfaction with own dental esthetics						
No	reference					
Yes	1.05	0.71	0.80, 1.38			
Interest in treatment						
No	reference					
Yes	0.77	0.05	0.58, 1.01	0.84	0.50	0.52, 1.38
CAT system						
ClearCorrect^TM^ aligner	reference			4.15	<0.01	1.72, 10.02
MODERN CLEAR system	16.52	<0.01	9.29, 29.39	69.94	<0.001	25.72, 190.18
Ortho Aligner	1.41	0.26	0.77, 2.56	6.06	<0.001	2.48, 14.79
Ideal Smile^®^ ALIGNER	2.19	0.01	1.25, 3.87	9.86	<0.001	3.87, 25.16
Orthocaps^®^ SLP 800	78.61	<0.01	28.58, 216.20	346.33	<0.001	109.05, 1099.87
Orthocaps^®^ DLP 460	7.11	<0.01	3.87, 13.08	32.29	<0.001	11.99, 86.96
Orthocaps^®^ DLP 580	16.52	<0.01	8.95, 30.49	78.25	<0.001	29.70, 206.11
No aligner	0.22	<0.01	0.09, 0.53	reference		

**Table 7:** Population average GEE results for the effect of the CAT system on acceptability and visibility.

CAT system	Acceptability			Attractiveness		
	Odds Ratio	***p*-value**	95% CI	Odds Ratio	***p*-value**	95% CI
ClearCorrect^TM^ aligner	13.45	<0.001	7.81, 23.19	34.13	<0.001	30.85, 37.41
MODERN CLEAR system	23.55	<0.001	12.23, 45.35	37.38	<0.001	34.16, 40.60
Ortho Aligner	22.85	<0.001	12.03, 43.39	37.59	<0.001	34.38, 40.81
Ideal Smile^®^ ALIGNER	18.86	<0.001	10.31, 34.52	38.37	<0.001	35.01, 41.74
Orthocaps^®^ SLP 800	reference			reference		
Orthocaps^®^ DLP 460	20.66	<0.001	11.10, 38.46	34.00	<0.001	30.76, 37.24
Orthocaps^®^ DLP 580	6.37	<0.001	4.10, 9.92	28.26	<0.001	25.37, 31.15

### ATTRACTIVENESS

**Figure 5:**
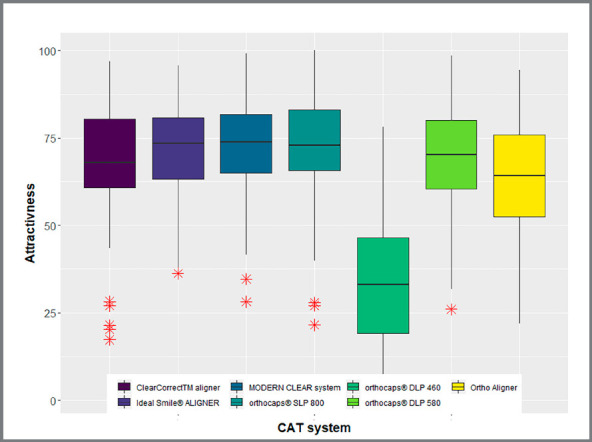
Predicted probabilities for willingness-to-pay per expertise status.

The distribution of attractiveness VAS scores per CAT system are tabulated in Table 5. Boxplots for attractiveness scores per CAT system are illustrated in Figure 5. On average, all but one CAT systems (i.e., Orthocaps^®^ SLP 800) were assigned moderate to high attractiveness scores, ranging from 63.04±17.44 to 73.15±15.05. There was a significant association between CAT system and attractiveness (*p*<0.001, Table 7). 

## DISCUSSION

This study may have direct implications in practice management and promotional strategies related to CAT systems. In general, the examined CAT systems received favorable esthetic responses regarding visibility, attractiveness and acceptability. Global esthetic superiority of a particular CAT system to competing products was not substantiated, and therefore aligner decision-making in daily practice should not be driven by such assumptions. 

As most participants expressed their willingness to pay up to 1,000 Euros more to receive CAT instead of traditional metallic appliances, offering this treatment technique may help orthodontists increase practice revenue and keep pace with patients’ needs for less visible appliances.^17^ This may also be of great interest for individuals seeking orthodontic treatment, as indicated by the high prevalence of previously treated participants keen on paying for CAT. Similarly, Rosvall et al.^15^ found that adults intended to pay an additional amount of 610 USD for CAT systems and lingual appliances. 

Notwithstanding the significant clinical benefits of CAT systems such as improved periodontal health indexes,^18^ shortened treatment duration and chair-time in mild-to-moderate cases,^19^ CAT is still considered not effective in controlling anterior extrusion, anterior buccolingual inclination, and rotation of rounded teeth^20^. This technical limitation of CAT, potentially familiar to dental professionals keeping up-to-date with the new literature, may explain why significantly more dental instructors declined CAT.

Dental expertise did not seem to be a significant predictor in rating aligner esthetics by first-year, sixth-year dental students and instructors. Unlike evidence supporting the substantially positive effect of clinical training on the assessment of facial and dental esthetics,^21^ longer experience in the dental field did not enable advanced year students or instructors to identify significantly more frequently the aligner images than beginner students. However, the present results are in line with reports on dental esthetics assessment, without significant differences between dentists and dental students.^22^ ^-^ ^24^

Visibility and acceptability responses as well as attractiveness VAS scores were not associated with orthodontic treatment history of the participants. Orthodontic patients may develop high valued esthetic awareness due to the increased attention paid during treatment appointments.^25^ ^,^ ^26^ The assumed higher esthetic standards of formerly treated individuals were neither confirmed by competence in recognizing CAT systems on the volunteer’s images nor by a tendency to assign higher attractiveness ratings.

Female participants were more skilled in identifying aligner presence and more willing to pay for CAT systems than males, but these sex differences did not reach statistical significance. Comparable preferences for facial, dental and smile esthetics between the sexes have been reported elsewhere among dental professionals.^21^ ^,^ ^27^ ^,^ ^28^

## STUDY LIMITATIONS

This study have some limitations. In accordance with similar studies^13^ ^-^ ^16^, CAT systems were examined in a volunteer with well-aligned teeth, not representing the average orthodontic patient. If this were not the case, probably tooth misalignment such as rotations and crowding could have compromised the appearance of the aligners and the appliance ratings. Technical parameters like aging and discoloring of the aligners were not considered in the current study design. From the practical point of view, CAT systems are not subjected to substantial esthetic changes during the recommended two-week wear in patients complying with oral hygiene and aligner cleaning instructions.^15^ To reinforce aligner retention and facilitate complex tooth movement, resin attachments are regularly used in CAT technique. Recent research shows that adults tend to favor clear aligners without attachments and ceramic brackets over clear aligners with multiple attachments.^29^ As CAT companies have developed different attachment shapes,^30^ investigation of the effect of attachment type on esthetics of several CAT systems would have presented methodological challenges.

## RECOMMENDATIONS FOR FUTURE RESEARCH

It would be useful to compare expert and lay groups, such as orthodontists and orthodontic residents, against adolescent and adult orthodontic patients or patients’ parents. The participants in this survey, as dental professionals, can be considered more trained in identifying deviation from the esthetic norms, in comparison to laypersons^23^. In addition to this, the strict esthetics standards of dentists may not coincide with patients’ perceptions.^28^ Finally, as this research focused entirely on subjective perceptions, the combined study of material properties and participants’ preferences is necessary to gain more insight into CAT esthetics. 

## CONCLUSIONS

» Esthetic perception of CAT systems by dental undergraduate students and instructors was overall favorable.
» Expertise status was significantly associated with willingness-to-pay for CAT, with instructors more frequently preferring fixed orthodontic appliances than students.
